# 

**Published:** 1886-09

**Authors:** 


### THE AMERICAN JOURNAL OF DENTAL SCIENCE.

PUBLISHED MONTHLY.

$2.50 PER ANNUM.

EDITED BY

F. J. S. GORGAS, M. D., D. D. S.

VOL. 20 THIRD SERIES. NO. 5.

BALTIMORE:

SNOWDEN & COWMAN.

LONDON:

TRUBNER & CO., 60 PATERNOSTER ROW.

PAYABLE IN ADVANCE.

